# Associations between initial dialysis access types and death from dialysis withdrawal in incident patients with kidney failure

**DOI:** 10.1093/ckj/sfaf024

**Published:** 2025-01-29

**Authors:** Jenny H C Chen, David W Johnson, Matthew A Roberts, Mark A Brown, Frank Brennan, Germaine Wong, Hicham Cheikh Hassan, Wing-Chi G Yeung, Alice Kennard, Christopher E Davies, Neil Boudville, Charmaine E Lok, Wai H Lim

**Affiliations:** Department of Renal Medicine, Wollongong Hospital, Wollongong, Australia; School of Medicine, University of Wollongong, Wollongong, Australia; Department of Kidney and Transplant Services, Princess Alexandra Hospital, Brisbane, Australia; Centre for Health Services Research, University of Queensland, Brisbane, Australia; The University of Queensland, Australasian Kidney Trials Network, Brisbane, Australia; School of Medicine, Monash University, Melbourne, Australia; Renal Service, Eastern Health, Melbourne, Australia; Department of Nephrology, St George Hospital, Sydney, Australia; School of Medicine, University of New South Wales, Sydney, Australia; Department of Nephrology, St George Hospital, Sydney, Australia; School of Medicine, University of New South Wales, Sydney, Australia; Sydney School of Public Health, University of Sydney, Sydney, Australia; Centre for Kidney Research, The Children's Hospital at Westmead, Westmead, Australia; Centre for Transplant and Renal Research, Westmead Hospital, Westmead, Australia; Department of Renal Medicine, Wollongong Hospital, Wollongong, Australia; School of Medicine, University of Wollongong, Wollongong, Australia; School of Medicine, Lebanese American University, Beirut, Lebanon; Department of Renal Medicine, Wollongong Hospital, Wollongong, Australia; School of Medicine, University of New South Wales, Sydney, Australia; Renal & Metabolic Division, The George Institute for Global Health, Sydney, Australia; School of Medicine, Australian National University, Canberra, Australia; Department of Nephrology, Canberra Hospital, Canberra, Australia; Australia and New Zealand Dialysis and Transplant Registry, South Australian Health and Medical Research Institute, Adelaide, Australia; Adelaide Medical School, University of Adelaide, Adelaide, Australia; Medical School, University of Western Australia, Perth, Australia; Department of Renal Medicine, Sir Charles Gairdner Hospital, Perth, Australia; Department of Medicine, Division of Nephrology, University Health Network, Toronto, Ontario, Canada; Faculty of Medicine, University of Toronto, Toronto, Ontario, Canada; Medical School, University of Western Australia, Perth, Australia; Department of Renal Medicine, Sir Charles Gairdner Hospital, Perth, Australia

**Keywords:** arteriovenous fistula, catheter, dialysis, mortality, withdrawal

## Abstract

**Background:**

Patients receiving haemodialysis via a central venous catheter (HD-CVC) have been shown to have an increased risk of all-cause mortality. It is unclear whether death from dialysis withdrawal is associated with the high mortality risk observed in patients initiated on HD-CVC.

**Methods:**

Using the Australia and New Zealand Dialysis and Transplant (ANZDATA) Registry, we examined the association between initial dialysis access [HD-CVC, haemodialysis via arteriovenous fistula (HD-AVF), and peritoneal dialysis (PD) via PD catheter (PD-PDC)] and death from dialysis withdrawal in adult patients starting dialysis in Australia between 2005 and 2022, analysed by time-stratified adjusted Cox regression with propensity score-matched cohorts.

**Results:**

Of 47 412 incident patients followed for a median of 2.65 years (interquartile range 1.19–4.87), 8170 (17%) died from dialysis withdrawal. Compared with patients initiated on HD-AVF, patients initiated on HD-CVC were more likely to experience death from dialysis withdrawal in the first 3 years after dialysis initiation, but not after 3 years [adjusted hazard ratios 2.43 (95% confidence interval 1.95–3.02), 2.06 (1.67–2.53), 1.57 (1.40–1.76), and 1.06 (0.97–1.15) for 0–6 months, >6–12 months, >1–3 years, and >3 years after dialysis initiation, respectively]. Comparison between patients initiated on HD-CVD and PD-PDC showed similar estimates. No difference in withdrawal risk was observed between patients initiated on HD-AVF and PD-PDC.

**Conclusions:**

Patients initiated on HD-CVC were twice as likely to experience early death from dialysis withdrawal compared with patients who had initiated dialysis with HD-AVF or PD-PDC. The increased risks diminished over time and were not observed after 3 years on dialysis.

KEY LEARNING POINTS **What was known:**

- Death from dialysis withdrawal is one of the most common causes of death in patients receiving maintenance dialysis.
- Patients initiated on haemodialysis via central venous catheter (HD-CVC) had greater all-cause mortality, compared with patients who initiated on haemodialysis via arteriovenous fistula (HD-AVF).
- Current literature assessing dialysis withdrawal in patients receiving dialysis via different types of dialysis access is limited.

**This study adds:**

- Patients initiated on HD-CVC were twice as likely to experience dialysis withdrawal in the first 12 months after dialysis initiation, compared with other types of dialysis access.
- The increased risk of dialysis withdrawal in patients initiated on HD-CVC diminished over time. There was no association observed between initial type of dialysis access and dialysis withdrawal 3 years post-dialysis initiation.

**Potential impact:**

- Recognizing the risk of early dialysis withdrawal in patients initiated on HD-CVD can better inform the shared decision-making process when selecting an appropriate treatment modality in patients with kidney failure.
- Delaying conversion from HD-CVC to HD-AVF or peritoneal dialysis (PD) via PD catheter may be appropriate in selected patients. Further studies are needed to assess the risks and benefits of early dialysis access conversion, especially in the first 12 months after dialysis initiation.
- In patients to be initiated on dialysis via HD-CVC, early involvement of kidney supportive care or palliative care teams may assist the shared decision-making in the selection of dialysis versus non-dialysis pathways and may alleviate symptoms associated with kidney failure and dialysis treatment which could potentially limit withdrawal from dialysis.

## INTRODUCTION

Dialysis withdrawal is one of the most common causes of death in patients receiving dialysis [1–3]. Reasons for dialysis withdrawal include life-limiting comorbidities, lack of dialysis access, and psychosocial preferences [4]. A previous Australian cohort study showed that the initial dialysis access type was associated with the highest risk of early dialysis withdrawal, more than age or comorbidities. Patients who commenced haemodialysis via a central venous catheter (HD-CVC) were twice as likely to experience dialysis withdrawal in the first year of dialysis initiation compared with patients who started haemodialysis via autologous arteriovenous fistula (HD-AVF) [5]. However, this association may be explained by differences in patient characteristics that are known to be associated with mortality [6]. The long-term association of the initial dialysis access type with dialysis withdrawal is also uncertain.

Traditionally, a ‘fistula first’ approach was the recommendation, based on the 2001 National Kidney Foundation Kidney Disease Outcomes Quality Initiative (KDOQI) Clinical Practice Guidelines [7]. However, the notion of ‘fistula first policy’ has been challenged in recent times, especially in elderly patients with limited life expectancy and high risks of arteriovenous fistula (AVF) dysfunction and complications [8–10]. As older and frailer patients are more likely to commence HD-CVC, they may also be more ambivalent regarding long-term dialysis [10, 11]. Moreover, prior observational studies have suggested that female patients and patients referred to a nephrologist just prior to dialysis initiation are more likely to opt for dialysis withdrawal [2, 5, 12–14]. A better understanding of the association between dialysis access types and dialysis withdrawal could help facilitate the shared decision-making process, especially at the time of dialysis modality selection and the subsequent end-of-life care [8].

The aims of this study were two-fold. First, we examined the associations between types of dialysis access with dialysis withdrawal. Second, we examined whether the association between dialysis access types and dialysis withdrawal was modified by age, sex, or late nephrologist referral.

## MATERIALS AND METHODS

### Study design and study cohort

This was a retrospective, registry-based, observational cohort study. Adult patients (aged 18 years and over) commencing maintenance dialysis for kidney failure for the first time in Australia between 1 January 2005 and 31 December 2022 were included using data from the Australia and New Zealand Dialysis and Transplant (ANZDATA) Registry. Patients from New Zealand were excluded due to the lack of local data on residential postcode and socioeconomic status. The follow-up period was censored at death, kidney transplantation, loss to follow-up, or 31 December 2022, whichever occurred first. The conduct of this study was approved by the University of Western Australia Human Research Ethics Committee (reference 021/ET000360), Australia. The study was conducted in accordance with Strengthening the Reporting of Observational Studies in Epidemiology (STROBE) guidelines [15].

### Data collection

Baseline demographic and dialysis characteristics at the time of kidney replacement therapy initiation were extracted from the ANZDATA Registry, including age, sex, ethnicity, body mass index (BMI), geographical location (urban, regional, and remote), socio-economic status (SES; measured by Index of Relative Socio-economic Advantage and Disadvantage using postcode and categorized into tertiles) [16], era (categorized as 2005–10, 2011–16, and 2017–22), smoking status, comorbid medical conditions (presence or absence of coronary artery disease, peripheral vascular disease, cerebrovascular disease, chronic lung disease, diabetes mellitus, and cancer), late nephrologist referral (defined as <3 months before dialysis initiation), primary cause of kidney failure (diabetic kidney disease, glomerulonephritis, hypertensive nephrosclerosis, cystic kidney disease, and other causes) and prior pre-emptive kidney transplantation. Baseline centre-level characteristics included transplant centre and centre size [calculated as the annual number of patients on incident haemodialysis, peritoneal dialysis (PD) or either at the time of dialysis initiation]. Centre size was divided into quartiles by patient number and grouped into three categories by combining the second and third quartiles. The dialysis centre was defined as the centre where dialysis was initiated but did not consider the potential transfer to alternative dialysis centre(s) over time.

### Exposure factor

Dialysis access type at the initiation of dialysis was categorized into three groups: HD-AVF (autologous AVF), HD-CVC (tunnelled and non-tunnelled CVC), and PD-PDC (PD catheter). Patients initiated on PD were assumed to have initiated dialysis with a PD catheter. Patients initiated on haemodialysis without dialysis access reported at first haemodialysis were excluded. Patients initiated on haemodialysis via arteriovenous graft (HD-AVG) were excluded in the primary analysis due to small sample size and potentially different patient phenotype compared with patients on HD-AVF. In the sensitivity analysis, patients initiated on HD-AVG were included in combination with HD-AVF as a single cohort. Initial haemodialysis access was defined as the access at first haemodialysis reported in the ANZDATA annual survey.

### Clinical outcomes

The primary outcome was death from dialysis withdrawal. Death from dialysis withdrawal included psychosocial reasons, life-limiting comorbid medical conditions (cardiovascular disease, cerebrovascular disease, peripheral vascular disease, or cancer), and dialysis access difficulties. The causes of withdrawal were pre-specified in the ANZDATA Registry survey [17].

### Statistical analysis

The baseline data were expressed as numbers (proportion) for categorical data, mean ± standard deviation (SD) for normally distributed continuous data, and median [interquartile range (IQR)] for non-normally distributed continuous data. The all-cause and withdrawal-related mortality rates were expressed as events [95% confidence interval (CI)] per 1000 patient-years. Patients initiated on HD-AVF, HD-CVC, and PD-PDC were matched 1:1 separately in pairs using the nearest neighbour within a calliper width of 0.01 of the propensity score with no replacement. Propensity scores were calculated using multivariable logistic regression matching age, sex, ethnicity, BMI, late nephrology referral, comorbid medical conditions (coronary artery disease, peripheral vascular disease, cerebrovascular disease, chronic lung disease, diabetes mellitus, and cancer), primary kidney disease, geographical location, era, and SES. These factors were selected because of the established associations with dialysis withdrawal and/or all-cause mortality in patients maintained on dialysis [5, 18, 19].

The associations between dialysis access and dialysis withdrawal for matched cohorts were examined using adjusted Cox proportional hazard (PH) regression via model building, expressed as hazard ratios (HRs) with 95% CI. The starting time in all models was time of dialysis initiation. The PH assumptions of all models were checked graphically by plotting the Schoenfeld residuals. As PH assumptions were violated in all models, the time period between dialysis initiation and outcome event was divided into 0–6 months, >6–12 months, >1–3 years, and >3 years. Collinearity between covariates was checked by a correlation matrix of coefficients. Model 1 was univariate analysis, and Model 2 included non-modifiable factors. Medical factors were added in Model 3, and social factors were added in Model 4. Cluster effects were examined in Model 5 using the γ-distributed Cox shared frailty model, considering initial dialysis centre as the cluster. Model 3 was considered as the final model due to the lack of association with social factors and the lack of cluster effects. Flexible parametric PH analyses were conducted using the method described by Royston and Parmar [20] and plotted as continuous adjusted HRs during the follow-up period, up to 10 years post-dialysis initiation.

Competing risk regression analyses were conducted using the method described by Fine and Gray [21], expressed as subdistribution hazard ratios (SHRs). Covariates included in the competing risk models were identical to those included in Model 3 of the Cox regression models. Kidney transplantation and causes of death other than dialysis withdrawal were considered as competing events. Considering the potential differences in dialysis access outcomes based on age, sex, and late referral, pre-specified two-way interactions between vascular access and these variables were examined *a priori* using Model 3. Sensitivity analysis was conducted combining HD-AVG and HD-AVF as a single group. Multiple comparisons were assessed using the Benjamini–Hochberg procedure with 95% false discovery rate (FDR) confidence intervals. Statistical evaluation was performed using SPSS version 28 (IBM, Armonk, NY, USA) and STATA version IC 15.1 (StataCorp, College Station, TX, USA) statistical programs. *P*-values <0.05 were considered statistically significant.

## RESULTS

**Figure 1:**
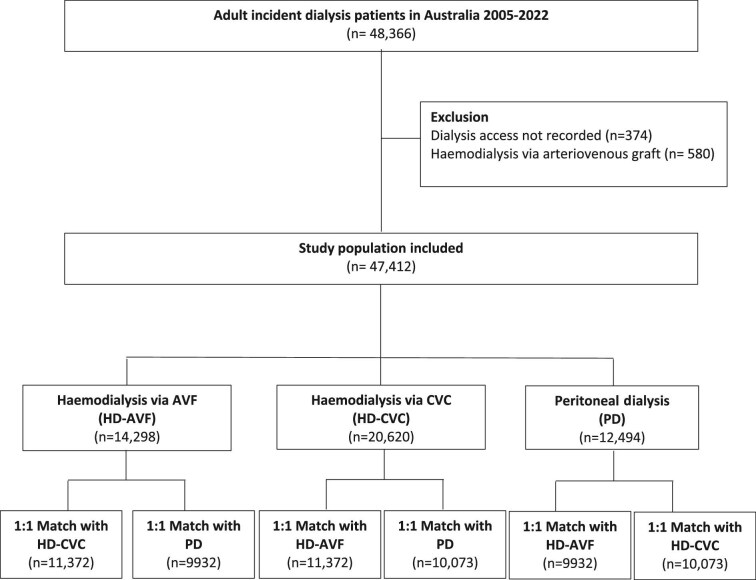
Patient flowchart for adult incident dialysis patients in Australia between 2005 and 2022.

The study cohort comprised 47 412 incident patients who commenced dialysis between 2005 and 2022 in Australia, after excluding 374 (0.8%) patients with missing initial dialysis access data and 580 (1.2%) patients who had initiated on haemodialysis using AVG (Fig. 1). The patient follow-up period was 165 126 person-years with median follow-up time of 2.65 (IQR 1.19–4.87) years. Among all incident patients, 14 298 (30%) patients commenced with HD-AVF, 20 620 (44%) patients commenced with HD-CVC, and 12 494 (26%) patients commenced with PD-PDC. The mean ± SD age at dialysis initiation was 61.1 ± 15.1 years. Compared with patients who were initiated on PD-PDC or HD-AVF, patients who were commenced on HD-CVC were more likely to experience late referral (35% vs 11% for PD-PDC and 6% for HD-AVF, *P *< 0.001) with a greater proportion with comorbid medical conditions at the time of dialysis initiation (Table 1). Supplementary Table 1 shows the baseline characteristics of the study cohort by the propensity score-matched pairs. Missing data are shown in Supplementary Tables 1 and 2.

**Table 1:** Baseline characteristics of adult patients commencing dialysis in Australia between 2005 and 2022.

	Descriptive statistics			
Characteristics	Haemodialysis via autologous AVF	Haemodialysis via CVC	Peritoneal dialysis	Overall
**Patient-level characteristics**				
Number of patients	14 298	20 620	12 494	47 412
Age starting dialysis (years, mean ± SD)	62.7 (14.0)	60.9 (15.8)	59.7 (15.2)	61.1 (15.1)
Men (*n*, %)	9419 (66)	12 486 (61)	7682 (62)	29 587 (62)
Ethnicity (*n*, %)				
Caucasians	10 456 (74)	14 325 (70)	8415 (68)	33 196 (71)
Asians	1090 (8)	1771 (9)	2148 (17)	5009 (11)
Indigenous^a^	1911 (13)	3418 (17)	1066 (9)	6395 (14)
Others	728 (5)	942 (5)	767 (6)	2437 (5)
BMI (mean ± SD)	29.8 (7.4)	28.5 (7.8)	27.3 (5.7)	28.6 (7.2)
BMI categories (*n*, %)				
Underweight	251 (2)	733 (4)	342 (3)	1326 (3)
Normal	3543 (25)	6507 (32)	4157 (34)	14207 (31)
Overweight	4368 (31)	6093 (30)	4402 (36)	14863 (32)
Obese	5895 (42)	6789 (34)	3457 (28)	16141 (35)
Dialysis modality (*n*, %)				
Peritoneal dialysis			12 494 (100)	12 94 (26)
Haemodialysis				
Facility haemodialysis	13 819 (97)	20 578 (99.8)		34 397 (73)
Home haemodialysis	479 (3)	42 (0.2)		521 (1)
Smoking status (*n*, %)				
Non-smoker	6690 (48)	9568 (47)	6311 (51)	22 569 (48)
Current smoker	1570 (11)	2784 (14)	1323 (11)	5677 (12)
Ex-smoker	5797 (41)	7980 (39)	4728 (38)	18 505 (40)
Comorbidities				
Chronic lung disease (*n*, %)	1705 (12)	3053 (15)	1069 (9)	5827 (12)
Coronary artery disease (*n*, %)	4637 (33)	7426 (36)	3284 (26)	15 347 (33)
Peripheral vascular disease (*n*, %)	2310 (16)	3964 (19)	1647 (13)	7921 (17)
Cerebrovascular disease (*n*, %)	1434 (10)	2391 (12)	1099 (9)	4924 (10)
Diabetes mellitus (*n*, %)				
Type 1	519 (4)	983 (5)	711 (6)	2213 (5)
Type 2	6817 (48)	9920 (48)	4905 (39)	21 642 (46)
Cancer (*n*, %)	1743 (12)	2688 (13)	1056 (9)	5487 (12)
Late nephrologist referral (*n*, %)	799 (6)	7215 (35)	1294 (11)	9308 (20)
Cause of kidney failure (*n*, %)				
Diabetic nephropathy	5573 (39)	8110 (40)	4280 (34)	17 963 (38)
Glomerular kidney disease	2584 (18)	3713 (18)	2983 (24)	9280 (20)
Hypertension nephrosclerosis	2048 (14)	2855 (14)	1817 (15)	6720 (14)
Familial/genetic kidney disease	1481 (10)	687 (3)	1063 (9)	3231 (7)
Tubulointerstitial disease	1310 (9)	1675 (8)	1121 (9)	4106 (9)
Others	1214 (9)	3431 (17)	1173 (9)	5818 (12)
Geographical location (*n*, %)				
Urban	9371 (66)	13 421 (66)	8739 (70)	31 531 (67)
Regional	3900 (28)	5411 (27)	3186 (26)	12 497 (27)
Remote	909 (6)	1602 (8)	499 (4)	3010 (6)
Socio-economic status (*n*, %)				
Low	4258 (30)	6333 (31)	3909 (32)	14 500 (31)
Mid	5444 (38)	7715 (38)	4707 (38)	17 866 (38)
High	4469 (32)	6379 (31)	3804 (31)	14 651 (31)
State/territory at dialysis initiation (*n*, %)				
New South Wales	3685 (26)	6027 (29)	4591 (37)	14 303 (30)
Queensland	2933 (21)	4156 (20)	2383 (19)	9472 (20)
Victoria	3770 (26)	4678 (23)	2937 (24)	11 385 (24)
Australian Capital Territory	301 (2)	518 (3)	162 (1)	981 (2)
South Australia	1316 (9)	1246 (6)	816 (7)	3378 (7)
Western Australia	1404 (10)	2573 (13)	1216 (10)	5193 (11)
Northern Territory	605 (4)	1017 (5)	159 (1)	1781 (4)
Tasmania	284 (2)	405 (2)	230 (2)	919 (2)
Era (*n*, %)				
2005–10	3957 (28)	6249 (30)	3318 (27)	13 524 (29)
2011–16	4702 (33)	6246 (30)	4244 (34)	15 192 (32)
2017–22	5639 (39)	8125 (40)	4932 (40)	18 696 (39)
Pre-emptive transplant (*n*, %)	78 (0.5)	125 (0.6)	91 (0.7)	294 (0.6)
**Centre-level characteristics**				
Number of centres	101	104	67	106
Transplant centre (*n*, %)	17 (17)	17 (16)	16 (24)	18 (17)
Centre size (median, IQR)				
Incidence dialysis patient/year	57 (31–101)	62 (38–101)	78 (45–113)	64 (38–106)
Incidence PD patient/year	13 (5–27)	15 (6–27)	22 (7–39)	16 (7–30)
Incidence HD patient/year	44 (24–70)	46 (28–71)	48 (31–70)	46 (28–70)

^a^Indigenous—Aboriginal and Torres Strait Islander, Polynesian.

### Mortality rates

**Figure 2:**
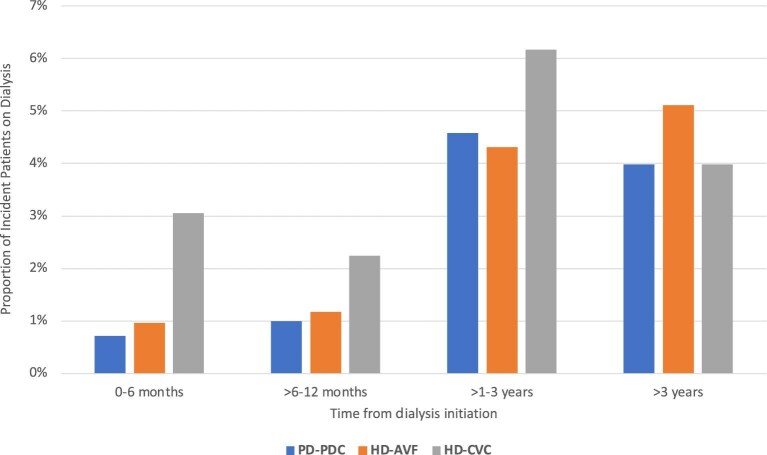
Proportion of incident patients on dialysis who withdrew from dialysis, stratified by initial dialysis access and time from dialysis initiation.

Among all incident patients, 22 838 (48%) patients died in the follow-up period, and 8170 (17%) died from dialysis withdrawal. The all-cause mortality rate was 138/1000 (95% CI 137–140/1000) patient-years, and the withdrawal-related mortality rate was 49/1000 (95% CI 48–51/1000) patient-years. Of the 8170 patients who died from dialysis withdrawal, 2382 (29%) were initiated via HD-AVF, 4007 (49%) were initiated via HD-CVC, and 1781 (22%) were initiated via PD-PDC. Figure 2 shows the proportion of incident patients on dialysis who withdrew from dialysis, stratified by dialysis vintage.

### Association between dialysis access and dialysis withdrawal: HD-CVC vs HD-AVF

**Figure 3:**
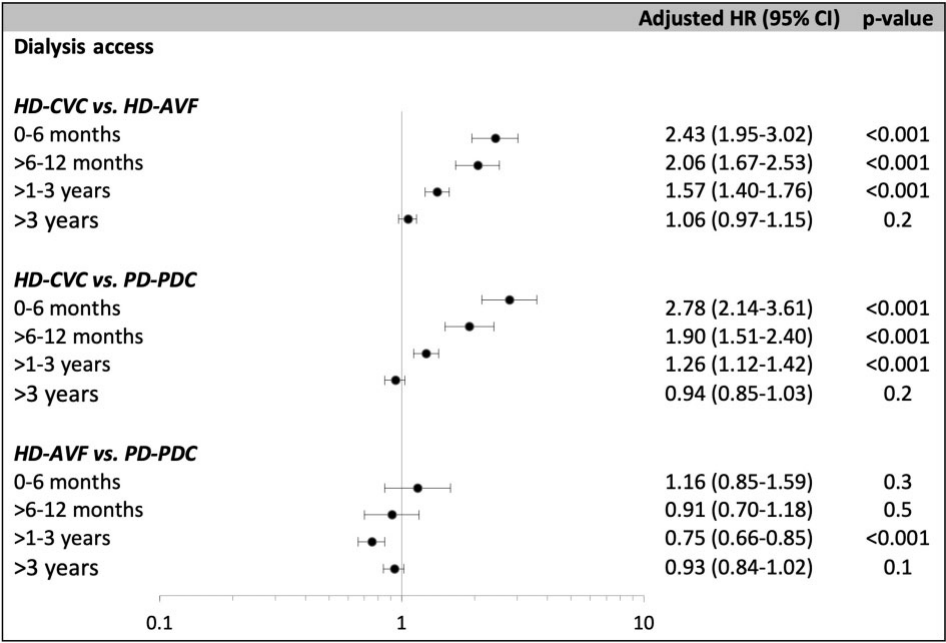
Cox regression analysis of the association between types of dialysis access and dialysis withdrawal, matched by propensity scores.

**Figure 4:**
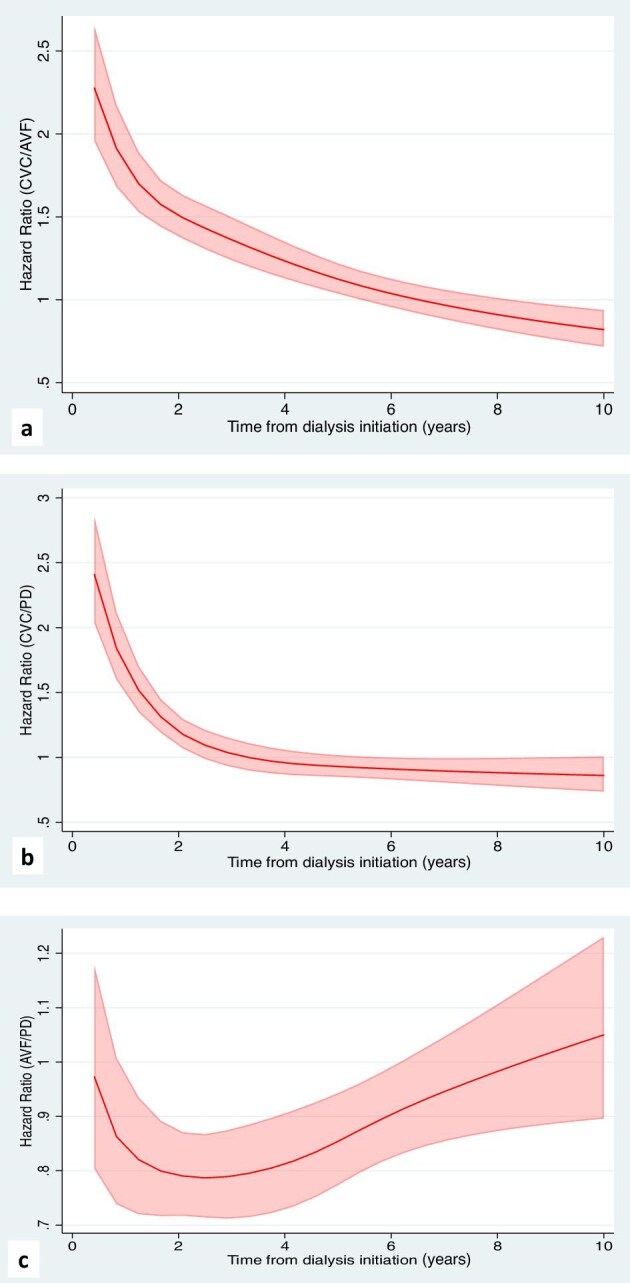
Continuous, adjusted cause-specific hazard ratios (solid lines; shaded areas are 95% CIs) up to 10 years after dialysis initiation, derived from the flexible parametric proportional hazards model. (**a**) Patients initiated on haemodialysis via central venous catheter (CVC) to patients initiated on haemodialysis via arteriovenous fistula (AVF). (**b**) Patients initiated on peritoneal dialysis (PD) to patients initiated on haemodialysis via central venous catheter (CVC). (**c**) Patients initiated on haemodialysis via arteriovenous fistula (AVF) to patients initiated on peritoneal dialysis (PD).

The propensity score-matched, paired cohort comprised 22 744 incident patients, with 11 372 pairs of patients commenced on HD-CVC and HD-AVF. Standardized percentage bias is shown in Supplementary Fig. 1a. Compared with patients who were initiated on HD-AVF, patients on HD-CVC were more likely to withdraw from dialysis in the first 3 years after dialysis initiation with reduced magnitude over time [Model 3 adjusted HR (aHR) for 0–6 months: 2.43, 95% CI 1.95–3.02; >6–12 months: 2.06, 1.67–2.53; >1–3 years: 1.57, 1.40–1.76]. No difference was observed after 3 years post-dialysis initiation (aHR 1.06, 0.97–1.15) (Table 2, Fig. 3). Analysis estimates for Models 3, 4, and 5 were similar. Figure 4a shows the continuous aHR for dialysis withdrawal comparing HD-CVC and HD-AVF, up to 10 years after dialysis initiation. Similar patterns were observed in the competing risk analyses (Supplementary Fig. 2).

**Table 2:** Propensity score-matched Cox regression analysis examining the associations between dialysis access and dialysis withdrawal.

	Model 1 HR (95% CI)	Model 2 HR (95% CI)	Model 3 HR (95% CI)	Model 4 HR (95% CI)	Model 5 HR (95% CI)
HD-CVC vs HD-AVF					
0–6 months	2.47 (1.98–3.08)	2.37 (1.90–2.96)	**2.43 (1.95–3.02)**	2.41 (1.93–3.01)	2.27 (1.75–2.95)
>6–12 months	2.04 (1.65–2.51)	2.00 (1.63–2.47)	**2.06 (1.67–2.53)**	2.06 (1.67–2.54)	2.00 (1.56–2.56)
>1–3 years	1.53 (1.36–1.72)	1.53 (1.36–1.72)	**1.57 (1.40–1.76)**	1.57 (1.40–1.77)	1.60 (1.44–1.87)
>3 years	1.00 (0.92–1.09)	1.04 (0.96–1.14)	**1.06 (0.97–1.15)**	1.07 (0.98–1.16)	1.07 (0.98–1.18)
HD-CVC vs PD-PDC					
0–6 months	2.89 (2.22–3.74)	2.70 (2.08–3.50)	**2.78 (2.14–3.61)**	2.74 (2.11–3.57)	2.83 (2.05–3.91)
>6–12 months	1.90 (1.51–2.39)	1.86 (1.48–2.35)	**1.90 (1.51–2.40)**	1.87 (1.48–2.36)	1.81 (1.37–2.39)
>1–3 years	1.25 (1.10–1.40)	1.25 (1.10–1.40)	**1.26 (1.12–1.42)**	1.25 (1.10–1.41)	1.22 (1.06–1.40)
>3 years	0.88 (0.80–0.96)	0.94 (0.85–1.03)	**0.94 (0.85–1.03)**	0.94 (0.85–1.03)	0.93 (0.83–1.03)
HD-AVF vs PD-PDC					
0–6 months	1.19 (0.87–1.63)	1.18 (0.86–1.62)	**1.16 (0.85–1.59)**	1.11 (0.81–1.53)	1.36 (0.92–2.01)
>6–12 months	0.93 (0.71–1.21)	0.92 (0.71–1.20)	**0.91 (0.70–1.18)**	0.89 (0.68–1.17)	0.92 (0.66–1.29)
>1–3 years	0.76 (0.66–0.86)	0.76 (0.66–0.86)	**0.75 (0.66–0.85)**	0.73 (0.64–0.84)	0.75 (0.64–0.88)
>3 years	0.90 (0.82–0.99)	0.94 (0.86–1.03)	**0.93 (0.84–1.01)**	0.92 (0.83–1.01)	0.93 (0.84–1.03)

Model 1: univariate.

Model 2: Model 1 + non-modifiable variables (age, gender, ethnicity).

Model 3: Model 2 + medical variables [late nephrology referral, body mass index, comorbid medical conditions (coronary artery disease, cerebrovascular disease, chronic lung disease, peripheral vascular disease, cancer, diabetes mellitus, primary kidney disease)].

Model 4: Model 3 + social variables (era, geographical location, socio-economic status, Australian state at time of dialysis initiation).

Model 5: Model 4 + centre-level characteristics (transplant centre, centre size).

Final model: Model 3 (bold).

### Association between dialysis access and dialysis withdrawal: HD-CVC vs PD-PDC

The propensity score-matched, paired cohort comprised 20 146 incident patients, with 10 073 pairs of patients commenced on HD-CVC and PD-PDC. Standardized percentage bias is shown in Supplementary Fig. 1b. Compared with patients who were initiated on PD-PDC, patients on HD-CVC were more likely to withdraw from dialysis in the first 3 years after dialysis initiation, with the greatest magnitude in the first 6 months (Model 3 aHR for 0–6 months: 2.78, 95% CI 2.14–3.61; >6–12 months: 1.90, 1.51–2.40; >1–3 years: 1.26, 1.12–1.42). No difference was observed after 3 years post-dialysis initiation (aHR 0.94, 0.85–1.03) (Table 2, Fig. 2). Analysis estimates for Models 3, 4, and 5 were similar. Figure 4b shows the continuous aHR for dialysis withdrawal comparing HD-CVC and PD-PDC, up to 10 years after dialysis initiation. Similar patterns were observed in the competing risk analyses (Supplementary Fig. 2).

### Association between dialysis access and dialysis withdrawal: HD-AVF vs PD-PDC

The propensity score-matched, paired cohort comprised 19 864 incident patients, with 9932 pairs of patients commenced on HD-AVF and PD-PDC. Standardized percentage bias is shown in Supplementary Fig. 1c. Compared with patients who were initiated via PD-PDC catheter, patients on HD-AVF were less likely to withdraw from dialysis between >1 and 3 years after dialysis initiation (Model 3 aHR 0.75, 95% CI 0.66–0.85). This was not apparent for other time periods (Table 2, Fig. 2). Analysis estimates for Models 3, 4, and 5 were similar. Figure 4c shows the flexible parametric survival plot showing the continuous aHR for dialysis withdrawal deaths comparing HD-AVF and PD-PDC, up to 10 years after dialysis initiation. Similar patterns were observed in the competing risk analyses (Supplementary Fig. 2).

### Interactions between dialysis access with sex, age or late referral for dialysis withdrawal

Of the 8170 patients who died from dialysis withdrawal, 4957 (61%) were men, and the mean (SD) age at time of dialysis initiation was 69.9 (±11.3) years. There was no interaction between dialysis access type and age or sex for dialysis withdrawal, across all time periods post-dialysis initiation. Inconsistent interaction was observed between dialysis access type and late referral for dialysis withdrawal (Table 3).

**Table 3:** Interactive effects between dialysis access with age, sex and late referral for dialysis withdrawal (Model 3).

	HD-CVC vs HD-AVF	HD-CVC vs PD -PDC	HD-AVF vs PD-PDC
Interaction between dialysis access and age (*P*_interaction_)			
0–6 months	0.61	0.85	0.62
>6–12 months	0.06	0.48	0.75
>1–3 years	0.53	0.75	0.82
>3 years	0.57	0.98	0.78
Interaction between dialysis access and sex (*P*_interaction_)			
0–6 months	0.57	0.79	0.57
>6–12 months	0.60	0.05	0.17
>1–3 years	0.82	0.69	0.52
>3 years	0.39	0.10	0.35
Interaction between dialysis access and late referral (*P*_interaction_)			
0–6 months	0.06	0.28	0.02
>6–12 months	0.03	0.11	0.61
>1–3 years	0.08	0.36	0.11
>3 years	0.12	0.60	0.52

### Sensitivity analysis

In the sensitivity analysis combining both AVF and AVG as a single group, the aHRs for dialysis withdrawal were similar to the primary analysis when compared with HD-CVC. There was no association observed comparing PD-PDC and HD-AVF/AVG for dialysis withdrawal, regardless of dialysis vintage (Supplementary Table 3). The FDR assessment showed wider confidence intervals, but the associations remained statistically significant (Supplementary Table 4).

## DISCUSSION

In this contemporaneous cohort study of incident patients with kidney failure in Australia, patients who were initiated on HD-CVC were more likely to experience dialysis withdrawal in the first 3 years after dialysis initiation, compared with patients on HD-AVF or PD-PDC. The magnitudes of the increased risks reduced exponentially over time. These observations provide a greater understanding of dialysis access and associated survival outcomes and offer guidance when choosing dialysis treatment, especially when life expectancy is limited.

Traditionally, AVF was the preferred vascular access for patients on haemodialysis with putative benefits [6, 8, 22–26]. Although the 2019 KDOQI guidelines did recognize the lack of evidence in mortality benefit for vascular access conversion in the first year of dialysis initiation, the guidelines still recommended that patients commenced on HD-CVC should convert to either an AVF or AVG if possible [8]. In our study, patients who were initiated on HD-CVC were twice as likely to withdraw from dialysis in the first 12 months of dialysis initiation compared with patients who were initiated on HD-AVF or PD-PDC. Survey studies from Canada and Ireland reported that patients on HD-CVC experienced a lower dialysis-related symptom burden and had better physical functioning scores compared with patients on HD-AVF [27, 28]. Delaying conversion from CVC to AVF/AVG/PDC may be appropriate in selected patients to prevent unnecessary medical procedures and clinical burden. Further studies assessing the harms and benefits of delaying dialysis access conversion are needed for the formulation of an end-stage kidney disease life plan and provision of individualized care, as suggested by the 2019 KDOQI guidelines [8].

Our study showed that the time between dialysis initiation and withdrawal for patients who commenced HD-CVC could be short, with the greatest hazard in the first 6 months after dialysis initiation. Although a time-limited dialysis trial for patients with uncertain prognosis may contribute to the early dialysis withdrawal [29], ANZDATA intends to only include patients receiving maintenance dialysis. Hence, patients with acute kidney injury on short-term dialysis were not reported to ANZDATA. During this uncertain period, advance care planning through the shared decision-making process is crucial. While on dialysis, patients may experience a significant symptom burden [30–33]. Addressing these symptoms may delay or prevent patients from dialysis withdrawal [30, 34]. Early involvement of a kidney supportive care (or palliative care) service may assist in the facilitation of the serious illness conversations, management of symptom burdens, and improvement of quality of life [34, 35]. Unfortunately, ANZDATA does not collect information on patient-reported outcomes to further assess the symptom burdens or quality of life.

Considering the likelihood of death from early dialysis withdrawal was significantly higher in patients who commenced HD-CVC, conservative kidney management (CKM, or non-dialysis pathway) may be a suitable alternative for certain patient groups [36]. Our study did not show any interactions of age and sex with dialysis access type in the association with dialysis withdrawal. Commencing HD-CVC may be a surrogate marker for frailty, psychosocial status, and health literacy, which are associated with increased mortality [37–39]. A previous ANZDATA study showed that older age, late nephrologist referral, and comorbid medical conditions were associated with dialysis withdrawal in the first 12 months after dialysis initiation [5]. More detailed studies on the predicting factors (including psychosocial wellbeing, socio-economic determinants, and severity of comorbid medical conditions) and their interactions with HD-CVC for early dialysis withdrawal will likely help identifying patients who may benefit from CKM, instead of dialysis initiation via CVC followed by early dialysis withdrawal. However, ANZDATA does not include patients receiving CKM to provide further insights for this patient group.

Mortality difference between PD and haemodialysis has constantly been debated despite numerous observational studies failing to provide a definitive answer [40–43]. Our study showed that withdrawal-related mortality differed significantly when comparing PD-PDC with HD-AVF versus HD-CVC, yet haemodialysis vascular access often was not differentiated in observational studies [8, 43]. In our study, patients who were commenced on dialysis via HD-AVF had a reduced risk of dialysis withdrawal 1–3 years after dialysis initiation, compared with patients commenced on PD-PDC. This may reflect PD discontinuation without transfer to haemodialysis. ANZDATA does not collect reasons for PD discontinuation in patients not transferred to haemodialysis; hence, such events were not examined further in our study.

There are several inherent limitations with observational registry data. First, ANZDATA collects dialysis access through the end-of-year survey reporting the initial and last dialysis access during the survey year. The duration of each dialysis access is not specified. Therefore, recall bias might occur, and the reported dialysis access might not be the primary dialysis access during the survey period. Second, dialysis withdrawal was reported by the treating centres, and only a single cause of death could be reported. The registry does not verify the accuracy of these causes, and misclassification bias of the cause of death is possible. Third, there are unmeasured and residual confounders which may be different between patients who were initiated on different dialysis access types. Information on severity of comorbid medical conditions, reasons for dialysis initiation and modality selection, psychosocial factors, functional status, and cognitive impairment is not collected by ANZDATA but might have modified the association between dialysis access and dialysis withdrawal.

In conclusion, initiating haemodialysis via CVC was associated with increased risks of dialysis withdrawal in the first 3 years of dialysis, compared with HD-AVF and PD-PDC, with the risks doubled in the first 12 months after dialysis initiation. Delaying the transition of dialysis access for patients initiated on HD-CVC and considering conservative kidney management with early involvement of kidney supportive care may minimize dialysis-associated psychosocial burdens and medical complications. Individualized treatment for kidney failure, including dialysis access choice, empowers clinicians and patients to balance the potential medical benefits and quality of life.

## Supplementary Material

sfaf024_Supplemental_Files

## Data Availability

The data underlying this article will be shared on reasonable request to the corresponding author.
