# Supplementary material for: Associations between initial dialysis access types and death from dialysis withdrawal in incident patients with kidney failure
Source: Clin Kidney J. 2025 Jan 29;18(3):sfaf024. doi: 10.1093/ckj/sfaf024 (PMC11879430; doi:10.1093/ckj/sfaf024)
Supplement: sfaf024_Supplemental_Files [file sfaf024_supplemental_files.zip › 1059 Supplementary material.docx]

**Supplemental Table 1.1:** Baseline characteristics of propensity score matched adult patients commencing haemodialysis via central venous catheter and haemodialysis via arteriovenous fistula in Australia between 2005 and 2022.

| Characteristics | HD-CVC vs. HD-AVF | | |  |
| --- | --- | --- | --- | --- |
|  | Haemodialysis via CVC | | Haemodialysis via AVF | Missing Data |
| Patient-Level Characteristics |  | |  |  |
| Number of patients | 11372 | | 11372 |  |
| Age starting dialysis  (year, mean±SD) | 62.7 (14.8) | | 62.3 (14.1) | 0 |
| Men (n, %) | 7374 (65) | | 7228 (64) | 0 |
|  |  | |  |  |
| Ethnicity (n, %) |  | |  | 0 |
| Caucasians | 8133 (72) | | 8121 (71) |  |
| Asians | 942 (8) | | 959 (8) |  |
| Indigenous^ | 1736 (15) | | 1727 (15) |  |
| Others | 561 (5) | | 565 (5) |  |
|  |  | |  |  |
| BMI (mean±SD) | 29.6 (7.8) | | 29.6 (7.4) | 0 |
| BMI Categories (n, %) |  | |  |  |
| Underweight | 218 (2) | | 230 (2) |  |
| Normal | 2983 (26) | | 3102 (27) |  |
| Overweight | 3583 (32) | | 3548 (31) |  |
| Obese | 4588 (40) | | 4492 (40) |  |
|  |  | |  |  |
| Dialysis Modality (n, %) | |  | | 0 |
| Peritoneal dialysis | -- | | -- |  |
| Haemodialysis |  | |  |  |
| Facility haemodialysis | 11346 (99.8) | | 11014 (97) |  |
| Home haemodialysis | 26 (0.2) | | 358 (3) |  |
|  |  | |  |  |
| Smoking Status (n, %) |  | |  | 173 |
| Non-smoker | 5291 (47) | | 5349 (47) |  |
| Current smoker | 1372 (12) | | 1325 (12) |  |
| Ex-smoker | 4632 (41) | | 4602 (41) |  |
|  |  | |  |  |
| Comorbidities |  | |  |  |
| Chronic lung disease (n, %) | 1502 (13) | | 1505 (13) | 0 |
| Coronary artery disease (n, %) | 4035 (35) | | 4054 (36) | 0 |
| Peripheral vascular disease (n, %) | 2041 (18) | | 2081 (18) | 0 |
| Cerebrovascular disease (n, %) | 1228 (11) | | 1252 (11) | 0 |
| Diabetes mellitus (n, %) |  | |  |  |
| Type 1 diabetes mellitus | 452 (4) | | 475 (4) | 0 |
| Type 2 diabetes mellitus | 5892 (52) | | 5870 (52) | 0 |
| Cancer (n, %) | 1413 (12) | | 1415 (12) | 0 |
|  |  | |  |  |
| Late Nephrologist Referral  (n, %) | 769 (7) | | 774 (7) | 0 |
|  |  | |  |  |
| Cause of Kidney Failure (n, %) |  | |  | 0 |
| Diabetic Nephropathy | 4906 (43) | | 4910 (43) |  |
| Glomerular Kidney Disease | 2130 (19) | | 2093 (18) |  |
| Hypertension Nephrosclerosis | 1691 (15) | | 1667 (15) |  |
| Familial/Genetic Kidney Disease | 529 (5) | | 535 (5) |  |
| Tubulointerstitial Disease | 1042 (9) | | 1006 (9) |  |
| Others | 1074 (9) | | 1161 (10) |  |
|  |  | |  |  |
| Geographical Location (n, %) |  | |  | 0 |
| Urban | 7538 (66) | | 7570 (67) |  |
| Regional | 3025 (27) | | 2984 (26) |  |
| Remote | 809 (7) | | 818 (7) |  |
|  |  | |  |  |
| Socio-economic Status (n, %) |  | |  | 0 |
| Low | 3490 (31) | | 3452 (30) |  |
| Mid | 4247 (37) | | 4291 (38) |  |
| High | 3635 (32) | | 3629 (32) |  |
|  |  | |  |  |
| State/Territory at Dialysis Initiation (n, %) | |  | | 0 |
| New South Wales | 3409 (30) | | 2904 (26) |  |
| Queensland | 2294 (20) | | 2346 (21) |  |
| Victoria | 2448 (22) | | 3005 (26) |  |
| Australian Capital Territory | 306 (3) | | 234 (2) |  |
| South Australia | 745 (7) | | 1026 (9) |  |
| Western Australia | 1403 (12) | | 1106 (10) |  |
| Northern Territory | 512 (5) | | 532 (5) |  |
| Tasmania | 255 (2) | | 219 (2) |  |
|  |  | |  |  |
| Era (n, %) |  | |  | 0 |
| 2005-2010 | 3251 (29) | | 3286 (29) |  |
| 2011-2016 | 3562 (31) | | 3494 (31) |  |
| 2017-2022 | 4559 (40) | | 4592 (30) |  |
|  |  | |  |  |
| Pre-emptive Transplant (n, %) | 90 (0.8) | | 48 (0.4) | 0 |

**Supplemental Table 1.2:** Baseline characteristics of propensity score matched adult patients commencing haemodialysis via central venous catheter and peritoneal dialysis in Australia between 2005 and 2022.

| Characteristics | HD-CVC vs. PD-PDC | | |  |
| --- | --- | --- | --- | --- |
|  | Haemodialysis via CVC | | Peritoneal Dialysis | Missing Data |
| Patient-Level Characteristics |  | |  |  |
| Number of patients | 10073 | | 10073 |  |
| Age starting dialysis  (year, mean±SD) | 60.5 (16.1) | | 60.4 (15.0) | 0 |
| Men (n, %) | 6176 (61) | | 6209 (62) | 0 |
|  |  | |  |  |
| Ethnicity (n, %) |  | |  | 0 |
| Caucasians | 7225 (72) | | 7185 (71) |  |
| Asians | 1297 (13) | | 1298 (13) |  |
| Indigenous^ | 935 (9) | | 1025 (10) |  |
| Others | 616 (6) | | 565 (6) |  |
|  |  | |  |  |
| BMI (mean±SD) | 28.3 (7.5) | | 27.6 (5.9) | 0 |
| BMI Categories (n, %) |  | |  |  |
| Underweight | 305 (3) | | 304 (3) |  |
| Normal | 3282 (33) | | 3316 (33) |  |
| Overweight | 3380 (34) | | 3364 (33) |  |
| Obese | 3106 (31) | | 3089 (31) |  |
|  |  | |  |  |
| Dialysis Modality (n, %) | |  | | 0 |
| Peritoneal dialysis | -- | | 10073 (100) |  |
| Haemodialysis |  | |  |  |
| Facility haemodialysis | 10048 (99.7) | | -- |  |
| Home haemodialysis | 25 (0.3) | | -- |  |
|  |  | |  |  |
| Smoking Status (n, %) |  | |  | 95 |
| Non-smoker | 5027 (50) | | 4950 (49) |  |
| Current smoker | 1200 (12) | | 1122 (11) |  |
| Ex-smoker | 3787 (38) | | 3965 (40) |  |
|  |  | |  |  |
| Comorbidities |  | |  |  |
| Chronic lung disease (n, %) | 954 (9) | | 988 (10) | 0 |
| Coronary artery disease (n, %) | 2975 (30) | | 3036 (30) | 0 |
| Peripheral vascular disease (n, %) | 1501 (15) | | 1537 (15) | 0 |
| Cerebrovascular disease (n, %) | 993 (10) | | 984 (10) | 0 |
| Diabetes mellitus (n, %) |  | |  | 0 |
| Type 1 diabetes mellitus | 522 (5) | | 624 (6) |  |
| Type 2 diabetes mellitus | 4428 (44) | | 4326 (43) |  |
| Cancer (n, %) | 943 (9) | | 981 (10) |  |
|  |  | |  |  |
| Late Nephrologist Referral  (n, %) | 1165 (12) | | 1250 (12) | 0 |
|  |  | |  |  |
| Cause of Kidney Failure (n, %) |  | |  | 0 |
| Diabetic Nephropathy | 3799 (38) | | 3804 (38) |  |
| Glomerular Kidney Disease | 2245 (22) | | 2207 (22) |  |
| Hypertension Nephrosclerosis | 1490 (15) | | 1508 (15) |  |
| Familial/Genetic Kidney Disease | 554 (6) | | 547 (5) |  |
| Tubulointerstitial Disease | 950 (9) | | 921 (9) |  |
| Others | 1035 (10) | | 1086 (11) |  |
|  |  | |  |  |
| Geographical Location (n, %) |  | |  | 0 |
| Urban | 7027 (70) | | 6966 (69) |  |
| Regional | 2631 (26) | | 2639 (26) |  |
| Remote | 415 (4) | | 468 (5) |  |
|  |  | |  |  |
| Socio-economic Status (n, %) |  | |  | 0 |
| Low | 3103 (31) | | 3109 (31) |  |
| Mid | 3835 (38) | | 3842 (38) |  |
| High | 3135 (31) | | 3122 (31) |  |
|  |  | |  |  |
| State/Territory at Dialysis Initiation (n, %) | |  | | 0 |
| New South Wales | 3122 (31) | | 3742 (37) |  |
| Queensland | 2011 (20) | | 1982 (20) |  |
| Victoria | 2308 (23) | | 2294 (23) |  |
| Australian Capital Territory | 267 (3) | | 136 (1) |  |
| South Australia | 666 (7) | | 671 (7) |  |
| Western Australia | 1180 (12) | | 925 (9) |  |
| Northern Territory | 276 (3) | | 136 (1) |  |
| Tasmania | 243 (2) | | 187 (2) |  |
|  |  | |  |  |
| Era (n, %) |  | |  | 0 |
| 2005-2010 | 2913 (29) | | 2895 (29) |  |
| 2011-2016 | 3190 (32) | | 3211 (32) |  |
| 2017-2022 | 3970 (39) | | 3967 (39) |  |
|  |  | |  |  |
| Pre-emptive Transplant (n, %) | 100 (1.0) | | 60 (0.6) |  |

**Supplemental Table 1.3:** Baseline characteristics of propensity score matched adult patients commencing haemodialysis via arteriovenous fistula and peritoneal dialysis in Australia between 2005 and 2022.

| Characteristics | HD-AVF vs. PD-PDC | | |  |
| --- | --- | --- | --- | --- |
|  | Haemodialysis via Autologous AVF | | Peritoneal Dialysis | Missing Data |
| Patient-Level Characteristics |  | |  |  |
| Number of patients | 9932 | | 9932 | 0 |
| Age starting dialysis  (year, mean±SD) | 61.2 (14.5) | | 61.5 (14.3) | 0 |
| Men (n, %) | 6346 (64) | | 6376 (64) | 0 |
|  |  | |  |  |
| Ethnicity (n, %) |  | |  | 0 |
| Caucasians | 7295 (73) | | 7353 (74) |  |
| Asians | 1046 (11) | | 1010 (10) |  |
| Indigenous^ | 990 (10) | | 990 (10) |  |
| Others | 601 (6) | | 579 (6) |  |
|  |  | |  |  |
| BMI (mean±SD) | 28.7 (7.1) | | 28.0 (5.9) | 0 |
| BMI Categories (n, %) |  | |  |  |
| Underweight | 220 (2) | | 227 (2) |  |
| Normal | 3031 (31) | | 2926 (29) |  |
| Overweight | 3571 (36) | | 3514 (35) |  |
| Obese | 3110 (31) | | 3265 (33) |  |
|  |  | |  |  |
| Dialysis Modality (n, %) | |  | | 0 |
| Peritoneal dialysis | -- | | 9932 (100) |  |
| Haemodialysis |  | |  |  |
| Facility haemodialysis | 9584 (96) | | -- |  |
| Home haemodialysis | 348 (4) | | -- |  |
|  |  | |  |  |
| Smoking Status (n, %) |  | |  | 118 (0.6) |
| Non-smoker | 4820 (49) | | 4822 (49) |  |
| Current smoker | 1134 (12) | | 1057 (11) |  |
| Ex-smoker | 3893 (40) | | 4020 (41) |  |
|  |  | |  |  |
| Comorbidities |  | |  |  |
| Chronic lung disease (n, %) | 933 (9) | | 967 (10) | 0 |
| Coronary artery disease (n, %) | 2868 (29) | | 2917 (29) | 0 |
| Peripheral vascular disease (n, %) | 1402 (14) | | 1437 (14) | 0 |
| Cerebrovascular disease (n, %) | 949 (10) | | 956 (10) | 0 |
| Diabetes mellitus (n, %) |  | |  |  |
| Type 1 diabetes mellitus | 426 (4) | | 519 (5) | 0 |
| Type 2 diabetes mellitus | 4174 (42) | | 4157 (42) |  |
| Cancer (n, %) | 963 (10) | | 971 (10) |  |
|  |  | |  |  |
| Late Nephrologist Referral  (n, %) | 723 (7) | | 676 (7) | 0 |
|  |  | |  |  |
| Cause of Kidney Failure (n, %) |  | |  | 0 |
| Diabetic Nephropathy | 3501 (35) | | 3563 (36) |  |
| Glomerular Kidney Disease | 2123 (21) | | 2073 (21) |  |
| Hypertension Nephrosclerosis | 1483 (15) | | 1468 (15) |  |
| Familial/Genetic Kidney Disease | 983 (10) | | 980 (10) |  |
| Tubulointerstitial Disease | 934 (9) | | 930 (9) |  |
| Others | 908 (9) | | 918 (9) |  |
|  |  | |  |  |
| Geographical Location (n, %) |  | |  |  |
| Urban | 6769 (68) | | 6733 (68) |  |
| Regional | 2701 (27) | | 2734 (28) |  |
| Remote | 462 (5) | | 465 (5) |  |
|  |  | |  |  |
| Socio-economic Status (n, %) |  | |  | 0 |
| Low | 3096 (31) | | 3059 (31) |  |
| Mid | 3772 (38) | | 3811 (38) |  |
| High | 3064 (31) | | 3062 (31) |  |
|  |  | |  |  |
| State/Territory at Dialysis Initiation (n, %) | |  | | 0 |
| New South Wales | 2635 (27) | | 3593 (36) |  |
| Queensland | 2011 (20) | | 1992 (20) |  |
| Victoria | 2658 (27) | | 2238 (23) |  |
| Australian Capital Territory | 198 (2) | | 137 (1) |  |
| South Australia | 950 (10) | | 694 (7) |  |
| Western Australia | 953 (10) | | 933 (9) |  |
| Northern Territory | 312 (3) | | 140 (1) |  |
| Tasmania | 2215 (2) | | 205 (2) |  |
|  |  | |  |  |
| Era (n, %) |  | |  | 0 |
| 2005-2010 | 2835 (29) | | 2829 (28) |  |
| 2011-2016 | 3297 (33) | | 3257 (33) |  |
| 2017-2022 | 3800 (38) | | 3846 (39) |  |
|  |  | |  |  |
| Pre-emptive Transplant (n, %) | 62 (0.6) | | 55 (0.6) | 0 |

**Supplemental Table 2:**

| Characteristics |  |  |
| --- | --- | --- |
|  | Missing Data | |
| Patient-Level Characteristics |  | |
| Total number of patients (n=47412) |  | |
| Age starting dialysis | 0 | |
| Sex | 0 | |
| Ethnicity (n, %) | 375 (0.9%) | |
| BMI (mean±SD) | 871 (2.1%) | |
| Dialysis Modality | 0 | |
| Smoking Status (n, %) | 661 (1.6%) | |
| Comorbidities |  | |
| Chronic lung disease (n, %) | 194 (0.5%) | |
| Coronary artery disease (n, %) | 197 (0.5%) | |
| Peripheral vascular disease (n, %) | 191 (0.5%) | |
| Cerebrovascular disease (n, %) | 196 (0.5%) | |
| Diabetes mellitus (n, %) | 181 (0.4%) | |
| Cancer (n, %) | 0 | |
| Late Nephrologist Referral | 477 (1.2%) | |
| Cause of Kidney Failure | 294 (0.7%) | |
| Geographical Location | 374 (0.9%) | |
| Socio-economic Status | 395 (1.0%) | |
| State/Territory at Dialysis Initiation | 0 | |
| Era | 0 | |
| Pre-emptive Transplant | 0 | |

**Supplemental Table 3**: Sensitivity analysis using propensity score matched Cox regression analysis examining the associations between dialysis access and dialysis withdrawal, combining haemodialysis via arteriovenous fistula and arteriovenous graft as a single group (Model 3).

|  | Sensitivity Analysis  Model 3^*^ HR (95%CI) | Primary Analysis  Model 3^*^ HR (95%CI) |
| --- | --- | --- |
| HD-CVC vs. HD-AVF/AVG | |  |
| 0-6 months  >6-12 months  >1-3 years  >3 years | 2.47 (1.98-3.07)  1.91 (1.56-2.34)  1.45 (1.30-1.63)  1.05 (0.97-1.14) | 2.43 (1.95-3.02)  2.06 (1.67-2.53)  1.57 (1.40-1.76)  1.06 (0.97-1.15) |
| HD-AVF/AVG vs. PD-PDC | |  |
| 0-6 months  >6-12 months  >1-3 years  >3 years | 1.01 (0.73-1.39)  1.02 (0.79-1.33)  0.92 (0.81-1.05)  0.92 (0.84-1.01) | 1.16 (0.85-1.59)  0.91 (0.70-1.18)  0.75 (0.66-0.85)  0.93 (0.84-1.01) |

HD-CVC – haemodialysis via central venous catheter; HD-AVF/AVG– haemodialysis via arteriovenous fistula/arteriovenous graft; PD-PDC – peritoneal dialysis via peritoneal dialysis catheter

* Model 3: dialysis access + non-modifiable variables (age, gender, ethnicity) + medical variables (late nephrology referral, body mass index, comorbid medical conditions (coronary artery disease, cerebrovascular disease, chronic lung disease, peripheral vascular disease, cancer, diabetes mellitus, primary kidney disease)

**Supplemental Table 4**: Adjusted 95% false discovery rate (FDR) confidence intervals using Benjamini-Hochberg procedure for multiple comparisons.

|  | Model 3  (FDR Adjusted 95%CI) |
| --- | --- |
| HD-CVC vs. HD-AVF |  |
| 0-6 months  >6-12 months  >1-3 years  >3 years | 2.43 (1.71-3.43)  2.06 (1.57-2.70)  1.57 (1.39-1.78)  1.06 (0.95-1.18) |
| HD-CVC vs. PD-PDC |  |
| 0-6 months  >6-12 months  >1-3 years  >3 years | 2.78 (2.08-3.71)  1.90 (1.47-2.46)  1.26 (1.10-1.45)  0.94 (0.84-1.05) |
| HD-AVF vs. PD-PDC |  |
| 0-6 months  >6-12 months  >1-3 years  >3 years | 1.16 (0.77-1.76)  0.91 (0.68-1.21)  0.75 (0.65-0.86)  0.93 (0.81-1.05) |

HD-CVC – haemodialysis via central venous catheter; HD-AVF – haemodialysis via arterio-venous fistula; PD-PDC – peritoneal dialysis via peritoneal dialysis catheter
