# Supplementary material for: Associations between initial dialysis access types and death from dialysis withdrawal in incident patients with kidney failure
Source: Clin Kidney J. 2025 Jan 29;18(3):sfaf024. doi: 10.1093/ckj/sfaf024 (PMC11879430; doi:10.1093/ckj/sfaf024)

	Adjusted SHR (95% CI)	p-value
**Dialysis Access**		
HD-CVC vs. HD-AVF		
0-6 months	2.37 (1.90-2.96)	<0.001
>6-12 months	2.04 (1.65-2.51)	<0.001
>1-3 years	1.28 (1.15-1.44)	<0.001
>3 years	1.01 (0.93-1.10)	0.8
HD-CVC vs. PD-PDC		
0-6 months	2.72 (2.10-3.53)	<0.001
>6-12 months	1.90 (1.50-2.40)	<0.001
>1-3 years	1.28 (1.13-1.44)	<0.001
>3 years	0.96 (0.88-1.06)	0.5
HD-AVF vs. PD-PDC		
0-6 months	1.17 (0.85-1.61)	0.3
>6-12 months	0.92 (.070-1.19)	0.5
>1-3 years	0.78 (0.68-0.89)	<0.001
>3 years	1.00 (0.91-1.10)	1.0

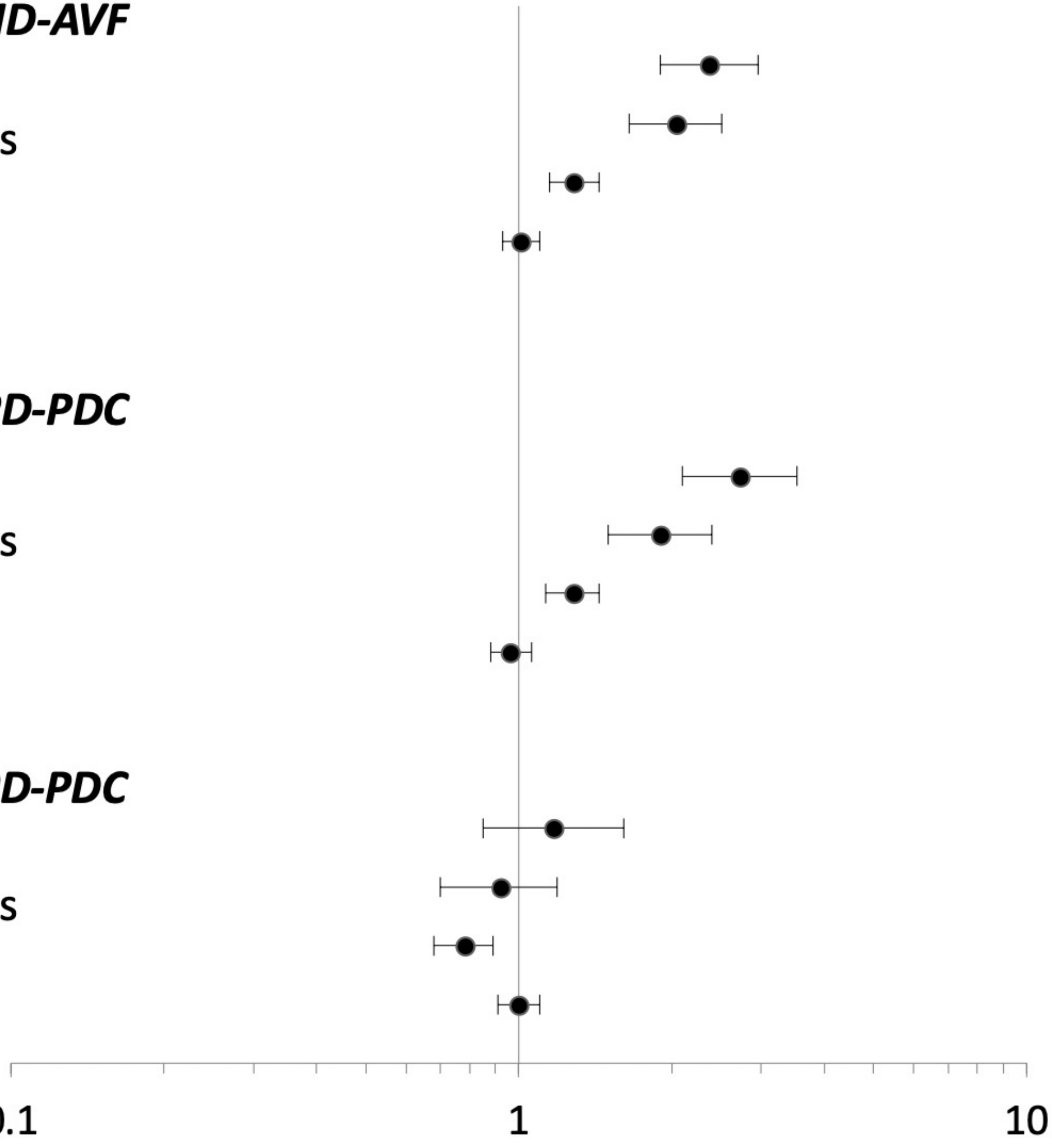

Supplement: sfaf024_Supplemental_Files [file sfaf024_supplemental_files.zip › SuppFigure2.pdf]
