# Supplementary figures and images for: Associations between initial dialysis access types and death from dialysis withdrawal in incident patients with kidney failure
Source: Clin Kidney J. 2025 Jan 29;18(3):sfaf024. doi: 10.1093/ckj/sfaf024 (PMC11879430; doi:10.1093/ckj/sfaf024)

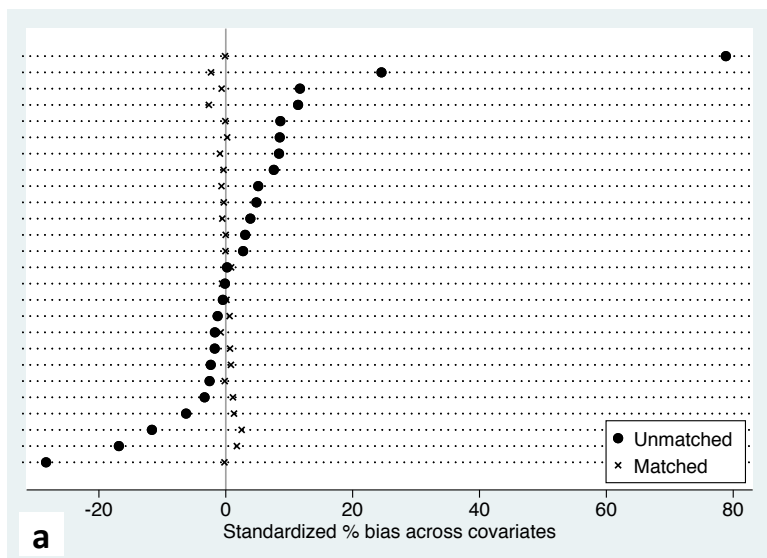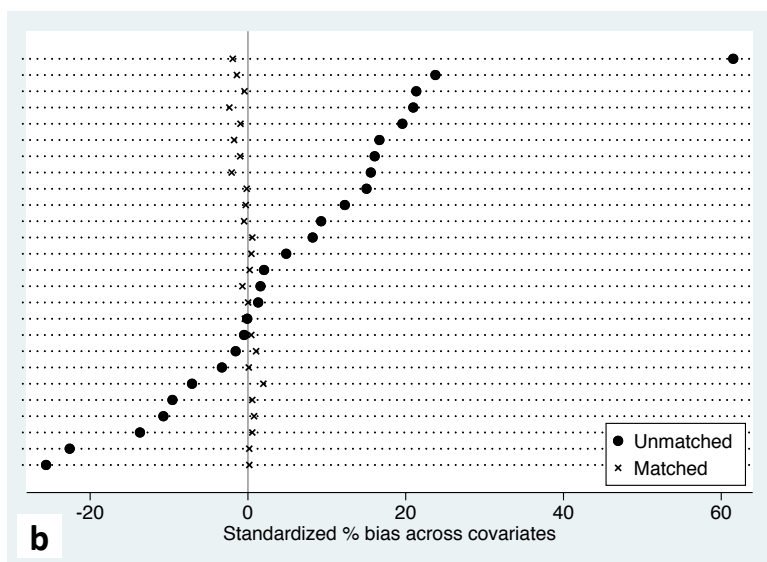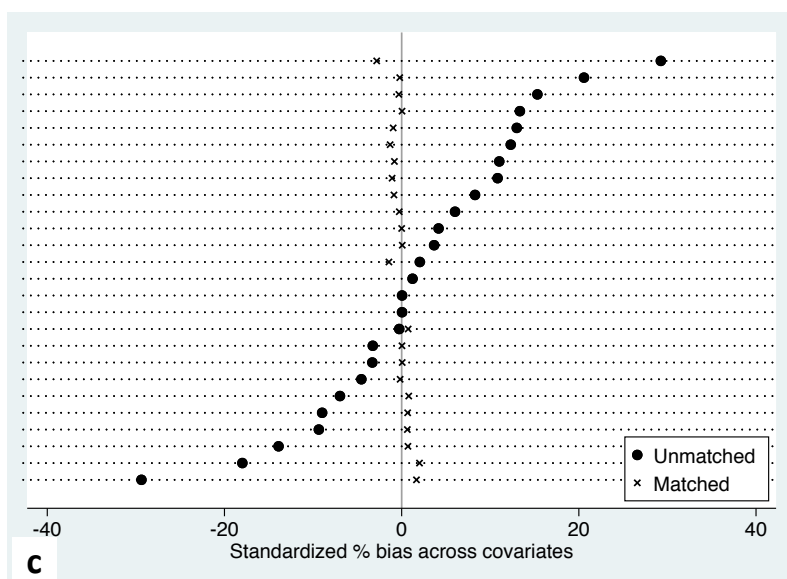

Supplement: sfaf024_Supplemental_Files [file sfaf024_supplemental_files.zip › SuppFigure1.pdf]
